# Microvessels Density in Uterine Leiomyosarcoma

**DOI:** 10.1155/2015/475305

**Published:** 2015-06-16

**Authors:** Marcin Bobiński, Wiesława Bednarek, Justyna Szumiło, Marek Cybulski, Grzegorz Polak, Jan Kotarski

**Affiliations:** ^1^1st Chair and Department of Gynaecological Oncology and Gynaecology, Medical University in Lublin, 16 Staszica Street, 20-081 Lublin, Poland; ^2^Chair and Department of Clinical Pathomorphology, Medical University in Lublin, Poland; ^3^Chair and Department of Biochemistry and Molecular Biology, Medical University in Lublin, Poland

## Abstract

Uterine leiomyosarcomas (LMS) are rare tumors typically presenting rapid growth and unfavorable outcome. Nowadays the results of uterine LMS treatment do not meet expectations. Angiogenesis is one of processes investigated to be target for future treatment. The aim of the research was to assess microvessels density (MVD) in tumor samples collected from 50 patients with histological confirmed uterine leiomyosarcoma and to investigate statistical relations between MVD, patients survival, and FIGO stage of tumor. The assessment was carried out using immunohistochemistry methods with anti-CD34 antibody. No significant difference in MVD between FIGO stages was observed. Furthermore, contrary to many other malignancies, we found no significant relation between MVD and patients overall and 2-year survival. Results obtained in the study suggest that processes on vascular mimicry and mesenchymal to epithelial transition (MET) may play important role in development of LMS. No statistical relation between MVD and survival leads to conclusion that not only angiogenesis but other mechanisms as well should be taken into consideration in planning future research.

## 1. Introduction

Angiogenesis is one of crucial processes in the development of various types of tumor. The correlation between this process and patients outcome has been investigated in many gynecological and nongynaecological malignancies [1, 2]. Recently many studies investigating potential role of angiogenesis in tumors diagnostics, prognostication, and therapy were released. During last few years many antiangiogenic agents were developed; therefore better understanding of angiogenesis and its role in tumors' biology seems to be necessary for the introduction of new therapeutic strategies.

Uterine sarcomas are very rare mesenchymal tumors; thus the number of reports concerning the biology is limited. The most common histological type of uterine sarcoma is leiomyosarcoma (LMS). These tumors usually present rapid growth and poor clinical outcome [3].

The activity of tumor's angiogenesis can be assessed by measuring microvessels density in its tissue. The CD34 glycoprotein is widely used as a marker of blood vessels' endothelial cells. Besides endothelium CD34 is also expressed in a membrane of steam cells, hemopoietic cells, and osteoclasts. Furthermore its expression was found in a few malignancies, that is, gastrointestinal stromal tumors, Kaposi sarcoma, and lymphoblastic leukemia [4].

The aim of this study was to assess microvessels density (MVD) in tumors tissues of uterine leiomyosarcomas using immunohistochemical staining with anti-CD34 antibody. Furthermore we aimed to indentify statistical relations of tumors MVD with overall survival (OS) and FIGO stage of the disease.

## 2. Materials and Methods

### 2.1. Patients and Tumor Samples

The retrospective study was performed using clinical data, follow-up, and paraffin samples of uterine leiomyosarcomas diagnosed among patients operated on in the 1st Department of Gynaecological Oncology and Gynaecology, Medical University in Lublin, Poland, from 2000 to 2013. Fifty patients were included to the study group. Mean age was 52,84 years (median: 51,50 y., SD: 12,36 y., min: 29 y., and max: 76 y.). FIGO stages among the patients are shown in Table 1. Histological diagnosis was than confirmed by two independent, experienced pathologists based on WHO criteria.

Forty-six patients (92%) underwent total hysterectomy and bilateral salpingo-oophorectomy (as first line or “second look” therapy); in two cases (4%) no surgical treatment was available and procedures were limited to collecting excisions, one patient (2%) had total hysterectomy without adnexectomy, and in one case (2%) retroperitoneal tumor was resected. Adjuvant treatment was provided to 9 patients, chemotherapy in 2 cases (4%), radiotherapy in 6 (12%), and radiochemotherapy in 1 case (2%).

### 2.2. Immunohistochemical Procedure

For immunohistochemical staining the most representative samples of tumors were chosen. Samples with extensive necrosis were excluded to avoid misinterpretation of vessel density, since areas close to necrosis are usually highly vascularized.

Tissue specimens have been cut into 3 *μ*m slides and fixed on silanized glass slides. Specimens were cleaned from paraffin and antigens were unmasked (using DAKO EnVision FLEX Retrieval Solution Low pH (50x) in DAKO PT Link, Pretreatment Module for Tissue Specimens (DAKO, Denmark)). Afterwards endogenous peroxidase was blocked by washing with hydrogen peroxide for 5 min. CD34 glycoprotein was marked by using specific antibodies (primary antibody: Monoclonal Mouse Anti Human CD34 Class II Clone QBEnd 10, secondary antibody: Dako EnVision+ System/HRP Labeled Polymer Anti Mouse). To gain colorful reaction, specimens were washed with DAB (diaminobenzidine) (DAKO, Denmark); afterwards cellular nuclei were stained with Meyer's hematoxylin.

### 2.3. MVD Assessment

**Figure 1:**
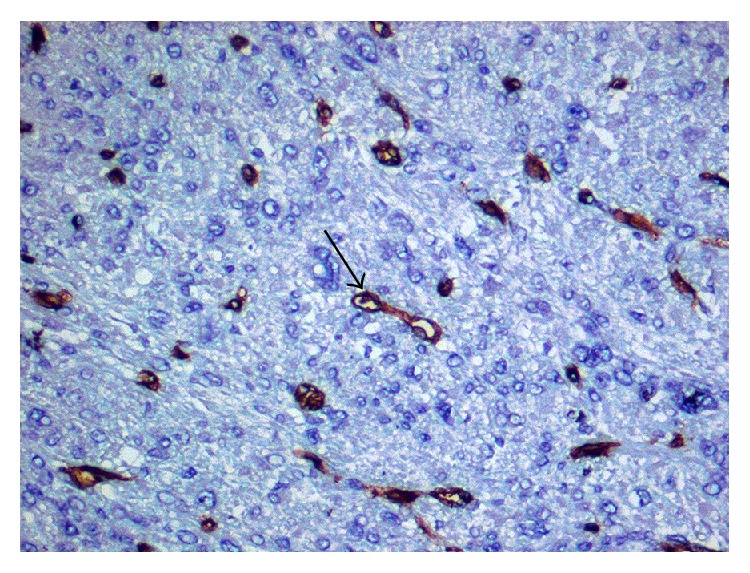
Colorful reaction with antibody against CD34 glycoprotein. Cellular nuclei stained with hematoxilin. The arrow shows CD34 positive microvessel. Magnification: 200x.

MVD was estimated by counting vessels containing CD34 positive cells in 10 HPF (high power field, magnification: 200x). MVD was expressed in absolute values as a number of CD34 positive cells in a field of 15,7 mm^2^.  Figure 1 presents microscopic view of LMS tissue stained with antibody against CD34.

### 2.4. Statistical Analysis

The distribution of results was tested with Kolmogorow-Smirnow with Lilliefors' modification and Shapiro-Wilk tests and assessed as nonnormal. To assess the correlation between MVD, FIGO, and overall survival Mann-Whitney test was used. To investigate relations between MVD and OS the group was divided into two subgroups, with high and low MVD, using median as the cut-off point. Differences in survival functions were analyzed with log-rank test (Mantel-Cox).

## 3. Results

CD34 positive vessels were observed in all the cases. MVD_CD34_ was ranged between 67 and 2917. Mean MVD_CD34_ was 929,26, median 766, and SD 592,03.

**Figure 2:**
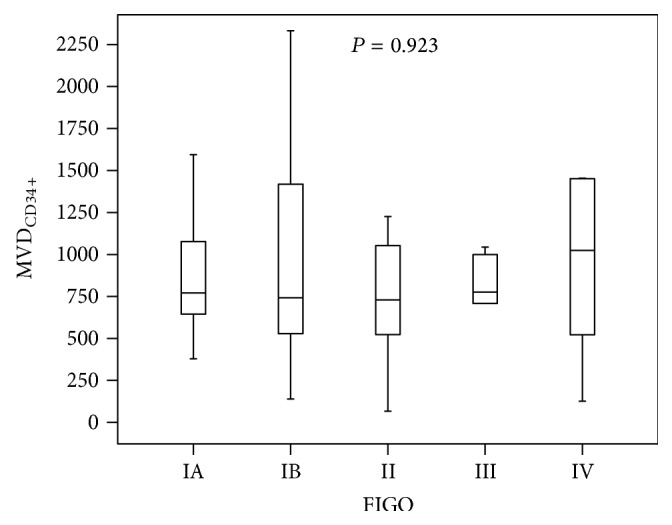
MVD_CD34_ depending FIGO stage.

Microvessels density in several FIGO stages is presented in Table 2 and Figure 2.

No significant difference in MVD_CD34_ between FIGO stages was observed (*P* = 0,923).

**Figure 3:**
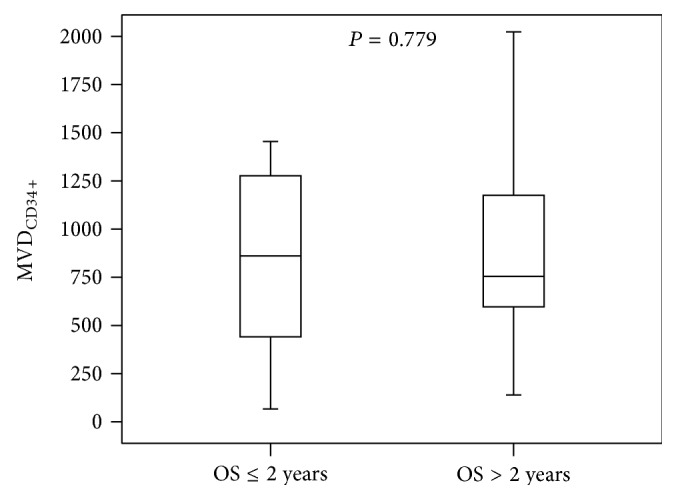
The MVD_CD34_ values in a groups of patients with OS longer and shorter than 2 years.

The MVD_CD34_ values in groups of patients with OS longer and shorter than 2 years are presented in Table 3 and Figure 3.

No significant differences in MVD_CD34_ in groups of patients with OS longer and shorter than 2 years were detected.

**Figure 4:**
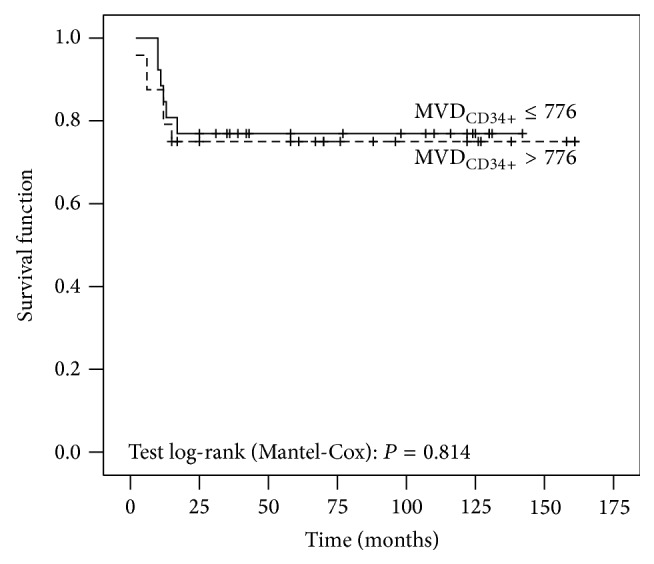
The Kaplan-Meyer survival functions of patients with uterine leiomyosarcoma by MVD_CD34_.

No significant differences in OS depending on MVD_CD34_ among patients with uterine leiomyosarcoma were detected (*P* = 0,814) (see Figure 4).

## 4. Discussion

### 4.1. MVD and Treatment Outcome in Uterine Sarcoma

Microvessels density is considered to be a marker of angiogenesis. The process of angiogenesis is crucial for tumors development. To asses MVD in tumors tissues many markers were used, that is, CD34, CD31, CD105, and von Willebrand factor [5–7]. However, it is still not proven which marker is the most suitable for such research. The undisputed advantage of glycoprotein CD34 is its high sensitivity and specificity, especially in endothelial cells staining.

In many malignancies MVD was found to be correlated with both overall and disease-free survival. In the studies carried on patients suffering from endometrial cancer, colorectal cancer, and lung cancer it was considered to be independent prognostic factor, where higher MVD was correlated with poorer outcome [8–10].

Taking into consideration typical limitations of sarcoma research such as small study groups, lack of prospective study, and not-standardized therapeutic strategies, only a few studies treating vascularity in uterine sarcoma have been published so far.

Poncelet et al. [6] investigated MVD in uterine leiomyosarcoma using antibodies against von Willebrand factor, CD34, and CD31 and concluded that MVD assessed only with antibodies against von Willebrand factor has prognostic value in this type of malignancy. Interestingly, lower MVD was correlated with poorer outcome. Interestingly there was no such relation with MVD assessed with antibodies against CD34 and CD31. Importantly, it occurs that MVD in sarcomas tissues was lower than in healthy myometrium. However, it is worth underlining that in cited research only 12 cases of LMS were analyzed.

Another research focused on MVD in leiomyosarcoma was conducted on a group of 66 patients by Avdalyan et al. [5]. They used antibodies against CD31 and concluded that MVD in tumor tissue does not affect survival but it does when it is assessed in peritumoral area.

In our research no statistical relation between MVD_CD34+_ and survival parameters was found. This fact combined with positive correlation with poorer prognosis and MVD in peritumoral area reported by Avdalyan et al. [5] may lead to the conclusion that there is a need to investigate other processes that may play an important role in the development of LMS. The role of vascularity in LMS tissue seems to be difficult to define. Recently, in a few centers sarcomas are investigated using xenografts and this method seems to be promising and may allow clarifying their role in angiogenesis in these rare tumors [11].

### 4.2. Angiogenesis as the Therapeutic Target in Uterine Sarcoma

Nowadays most of drugs used in chemotherapy express their activity by blocking cells division or inducing apoptosis in dividing cells. These therapies are efficient against cells that are dividing quickly, but it is widely known that many tumors (e.g., uterine sarcomas) have heterogenic histology and include, for example, steam like cells (cancer cells that express some features of steam cells, i.e., ability for self-renewal) with quite stable cell cycle that divide very seldom (tumorigenic cells) [12]. The presence of such cell populations in tumors' tissues is considered to be responsible for presence of recurrence.

Tumor development depends on oxygen and other substances' supply. This fact leads to concept of using inhibition of angiogenesis as antitumor therapy.

Interestingly many old, well-known drugs were found to have antiangiogenic activity (i.e., acetylsalicylic acid, thalidomide, and gold based antirheumatic drugs) [13].

During last few years many interesting ideas of antiangiogenic therapy were introduced. A few trials were conducted with uterine LMS as well.

In the phase II trial investigated activity of thalidomide in LMS it was concluded that it has no antitumor activity in this type of malignancies [14].

Acetylsalicylic acid (aspirin) widely using cyclooxygenase (COX) inhibitor is another agent that is tested to assess its antiangiogenic activity. The mechanism of COX inhibitors influence on angiogenesis is based on decreasing expression of proangiogenic factors such as VEGF-A and VEGF-C in tumor cells. Promising results were achieved using high dose of aspirin to inhibit growth of sarcoma cells cultures among mice [15]. In the research cited above except from inhibition of tumors growth lower MVD was noted as well.

Among novel antiangiogenic agents sunitinib was considered to be one of the most promising agents. Sunitinib is multitarget tyrosine kinase inhibitor that expresses activity against, for example, VEGF receptors, PDGF receptors, and stem cell factor receptor (KIT). Molecules mentioned above play an important role in tumors angiogenesis and development. Unfortunately, in latest research sunitinib was considered to be inactive in uterine LMS [16].

Trials aimed to assess activity of antiangiogenic drugs in LMS mostly have not met the expectations. The extrapolation of these conclusions may support results obtained in the presenting study.

The fact that MVD does not affect OS in uterine sarcoma may lead to deduction that LMS present special features that allow them to develop in a different way than other malignancies where relation between MVD and OS was observed.

This observation may be explained by phenomenon of “vascular mimicry” and “mesenchymal to epithelial transition” (MET).

### 4.3. Vascular Mimicry in LMS

Vascular mimicry is the formation of vascular-like structures but unprovided with endothelial cells. Walls of these structures are built of tumor cells that are suspected to present some antigens typical for endothelial cells (i.e., CD31). Interestingly, red blood cells have the ability to pass though mimicry structure similarly to passing through microvessels [17]. The presence of mimicry was observed in a few types of mesenchymal tumors, for example, rhabdomyosarcomas, esophageal stromal tumors, and it was linked with poor prognosis [18].

The process of vascular mimicry is still not fully understood and its role in development of LMS remains unclear. However, the lack of difference in MVD between tumors with various prognosis and clinical stages demands explanation and investigating presence of mimicry in this tumor seems to be promising way of further research.

### 4.4. MET in Sarcomas

Another recently described process that may be suspected to play a role in development of LMS' vascularity is “mesenchymal to epithelial transition.” Simplifying, it is the process of acquiring by mesenchymal tumor cells characteristics typical for epithelial cells. The presence of this process in LMS was already noted [19].

Possible ability of LMS cells to present endothelial-like phenotype could be important in understanding biology of these tumors. Glycoprotein CD34 is the one of epithelial markers that were observed among mesenchymal cells that undergone MET [20].

Assuming that at least part of tumors cells is able to differentiate into epithelial-like cells may explain its resistance for antiangiogenic therapy. This assumption allows supposing that even if its vascularity is affected by therapy, tumor has ability to build new endogenous vascular system to supply itself with necessary substances.

## 5. Conclusions

The fact that no relevance between MVD, OS, and FIGO stage exists in uterine sarcoma leads to conclusion that process of angiogenesis in these rare tumors demands further research. The efficiency of experimental antiangiogenic therapy remains unsatisfactory. Vascular mimicry and MET may play important role in these tumors and may occur promising prognostic factors and therapeutic targets. Future research on LMS should take into consideration possibility of presence of multipotential, steam-like tumors.

## Figures and Tables

**Table 1:** FIGO stages among patients.

FIGO stage	Number of patients
IA	10
IB	23
II	6
III	5
IV	6

**Table 2:** MVD_CD34 _ depending FIGO stage.

	*N*	Mean value ± SD	Median	Min–max	*P*
FIGO IA	10	849,40 ± 342,75	771,50	379–1594	0,923
FIGO IB	23	1059,74 ± 751,89	742,00	139–2917	
FIGO II	6	721,17 ± 411,44	729,00	67–1226	
FIGO III	5	733,40 ± 361,70	776,00	139–1044	
FIGO IV	6	933,50 ± 529,71	1024,00	126–1454	

**Table 3:** MVD_CD34_ values in a groups of patients with OS longer and shorter than 2 years.

	*N*	Mean ± SD	Median	Min–max	*P*
Survival ≤ 2 years	14	818,21 ± 491,71	861,00	67–1454	0,779
Survival > 2 years	36	972,44 ± 627,73	754,50	139–2917	
